# An Anti-inflammatory microRNA Signature Distinguishes Group 3 Innate Lymphoid Cells From Natural Killer Cells in Human Decidua

**DOI:** 10.3389/fimmu.2020.00133

**Published:** 2020-02-06

**Authors:** Andrea Pelosi, Claudia Alicata, Nicola Tumino, Tiziano Ingegnere, Fabrizio Loiacono, Maria Cristina Mingari, Lorenzo Moretta, Paola Vacca

**Affiliations:** ^1^Immunology Research Area, IRCCS Bambino Gesù Pediatric Hospital, Rome, Italy; ^2^Immunology Operative Unit, Department of Integrated Oncological Therapies, IRCCS Policlinico San Martino Hospital, Genoa, Italy; ^3^Department of Experimental Medicine, School of Medical and Pharmaceutical Sciences, University of Genoa, Genoa, Italy; ^4^Center of Excellence for Biomedical Research, University of Genoa, Genoa, Italy

**Keywords:** decidua, innate lymphoid cells, NK cells, ILC3, microRNA

## Abstract

Innate lymphoid cells (ILCs) are a heterogeneous subset of lymphocytes deeply implicated in the innate immune responses to different pathogens, in lymphoid organogenesis and in the maintenance of tissue homeostasis. Group 3 innate lymphoid cells (ILC3) have been detected in human decidua, where they play a role in the early inflammatory phase favoring implantation and tissue remodeling as well as in the subsequent regulatory phase preventing fetal rejection and supporting neoangiogenesis. A balance between inflammation and immune tolerance is required to maintain pregnancy, thus maternal immune system must be controlled by finely tuned mechanisms. microRNAs (miRNAs) are small non-coding RNAs with important regulatory roles in immune cells, but their function in decidual ILC3 (dILC3) and in decidual NK (dNK) cells is still undefined. Here, we examined the miRNome by microarray in these cells during the first trimester of pregnancy and compared with miRNA profiles of peripheral blood NK (pbNK) cells from pregnant women. We show that distinct miRNA profiles could clearly distinguish dILC3 from NK cells. Correlation analyses revealed that dNK and pbNK miRNome profiles are more similar to each other as compared to dILC3. In particular, we identified 302 and 279 mature miRNAs differentially expressed in dILC3 as compared to dNK and pbNK, respectively. The expression of *miR-574-3p* and the *miR-99b/let-7e/miR-125a* miRNA cluster resulted the most increased in dILC3. Remarkably, gene ontology analysis and pathway enrichments of miRNA targets revealed an involvement of these miRNAs in the promotion of anti-inflammatory responses. In agreement to this finding, we also found a higher expression of the anti-inflammatory *miR-146a-5p* in dILC3 with respect to NK cells. Overall, our data identified specific miRNA signatures distinguishing dILC3, dNK, and pbNK cells. Our data suggest the existence of a tight epigenetic control mediated by miRNAs in dILC3, potentially acting as a brake to prevent exaggerated inflammatory responses and to maintain the immune homeostasis in the early phases of pregnancy.

## Introduction

Innate lymphoid cells (ILCs) are a heterogeneous subset of lymphocytes characterized by the lack of an antigen-specific receptor. They represent the innate counterpart of helper T lymphocytes and are deeply implicated in the innate immune responses to pathogens, in lymphoid organogenesis and in the maintenance of tissue homeostasis (1). According to the recent advances on ILC biology and on their developmental trajectories, the classification of ILCs currently comprises five groups: NK cells, ILC1, ILC2, ILC3, and lymphoid tissue-inducers (LTi) cells (1).

The decidua is a transient maternal structure in the uterine tissue derived from the differentiation of the endometrial lining in preparation of pregnancy. As a maternal interface to the embryo, the decidua represents an important functional platform ensuring the exchange of nutrients, gas and catabolites as well as the protection of the embryo from the maternal immune system and the promotion of a finely controlled tissue remodeling for the invasion of trophoblast (2). The decidua contains a large number of maternal immune cells, the function of which is only partially understood. Major populations comprise innate immune cells, such as macrophages and ILCs, which are believed to establish a balance between defenses against pathogens and tolerance of the embryo (2).

Collectively, several findings in murine and human maternal compartments would suggest an important role of ILCs in both the initiation and maintenance of pregnancy (3). During the first trimester of pregnancy, NK cells reach 40–70% of total lymphocytes present in decidua representing the main lymphoid population and displaying unique phenotypic and functional features (4). Remarkably, decidual NK (dNK) are mainly characterized by a regulatory function displaying more pronounced functional characteristics favoring immune tolerance, necessary to ensure successful pregnancy (5, 6), whereas peripheral blood (pb) NK cells are characterized by cytolytic activity and production of cytokines such as IFN-γ and TNF (7, 8). Furthermore, dNK cells also play a role in immunity to infections (9). Among the others ILCs subsets, ILC3s have been the most extensively studied in human decidua (10, 11). Similarly to LTi cells, ILC3s are lineage negative (Lin^−^) CD127^+^ lymphoid cells relying on RORγt for their development (11). In humans, besides LTi cells, two subsets of ILC3 were identified on the basis of the cell surface expression of NKp44 and classified as Natural Cytotoxicity Receptor negative (NCR^−^) and NCR^+^ ILC3. *In utero*, NCR^+^ ILC3 population is predominant in humans (3). They have been identified in human decidua since the first trimester of pregnancy and they can produce GM-CSF, IL-22, and IL-8 cytokines (11). Recently, it has been reported that NCR^+^ ILC3 are involved in the recruitment and survival of neutrophils in decidua, suggesting a role in the early phases of pregnancy (10). However, also due to their relatively recent identification, a clear knowledge of ILC3 role in human decidua is far to be fully elucidated.

microRNAs (miRNAs) are an important class of single strand small non-coding RNAs (19–24 nucleotides). They mainly act as negative regulators of gene expression by binding to partially complementary sequences in the 3′ untranslated region of specific target messenger RNAs, resulting in their degradation or inhibition of their translation (12). Several studies have revealed a relevant role of miRNAs in NK cell biology, such as in the regulation of NK cell development, activation and effector functions (13–16). According to their different functions and phenotypes in specific tissues, human NK cells from different cell compartments exhibited specific miRNA signatures (14, 17). However, the miRNA content and regulatory function in other human ILC subsets have been poorly investigated. In particular, the miRNA expression in decidual ILC3 (dILC3), which play an important role during healthy pregnancy, is currently unknown.

In this study, we profiled 2,578 mature miRNAs in both dILC3, dNK, and pbNK cells and identified the miRNAs differentially expressed by these populations. The validation analysis confirmed that *miR-574-3p, miR-146a-5p, miR-99b-5p, let-7e-5p*, and *miR-125a-5p* expression is strikingly increased only in dILC3, whereas *miR-181b-5p, miR-486-5p* and *miR-151a-5p* expression is increased in pbNK cells as compared to dILC3 and dNK cells. Furthermore, miRNA targets analysis allowed us to identify a restricted signature of miRNAs with regulatory functions in the control of inflammatory responses in human decidua.

## Materials and Methods

### Human Samples

We collected human decidua (*n* = 13) and peripheral blood (*n* = 4) samples from singleton pregnancies of mothers at 9–12 weeks of gestation requesting termination of pregnancy for social reasons at AOU San Martino-IST (Genoa, Italy). Peripheral blood samples were obtained from 4 to 13 patients enrolled for the decidua samples collection. Exclusion criteria were HIV and HCV infected patients. Non-pregnant female controls were collected from volunteer blood donors (*n* = 4) admitted to the blood transfusion service of IRCCS Bambino Gesù Pediatric Hospital. Tonsils were obtained from female patients (*n* = 3) undergoing tonsillectomy due to recurrent tonsillitis at the Giannina Gaslini Institute (Genova, Italy). The relevant institutional review boards approved the study and all patients gave their written informed consent according to the Declaration of Helsinki.

### ILCs Isolation

Cell suspensions from decidual and tonsil tissues were obtained with GentleMacs dissociator (Miltenyi Biotech, Bergisch Gladbach, Germany) as previously described (18). Decidua infiltrating lymphocytes (DILs), tonsil lymphocytes and peripheral blood mononuclear cells from blood samples were isolated after density gradient centrifugation over Ficoll Lympholyte®-H Fycoll (Cederlane, Burlington, Canada). pbNK cells (purity >90% for all the samples; mean: 95%) were obtained with RosetteSep^TM^ human NK cell enrichment cocktail (StemCell Technologies; Vancouver, Canada). For decidual and tonsil ILC isolation, cell sorting was performed using a multiparametric gating strategy of CD45^+^/live DILs that allows to identify different ILC subsets (11). Subsequently, CD45^+^/live lymphocytes were gated as Lineage negative (Lin^−^: CD19^−^, CD14^−^, CD3^−^) cells and CD127^−^ (*bona fide* NK cells) or CD127^+^ cells (*bona fide* helper ILC). These subsets were further analyzed for the expression of CD117 and NKp44. The helper ILCs contain cells that expressed CD117 and NKp44 (this subset is referred to as NCR^+^ILC3) that also were CD56^+^ and CD94^−^ (markers allowing a more precise identification of ILC3 subsets). On the other hand, NK cell subset (CD127^−^CD117^low/−^) expressed CD56 and CD94. All samples were analyzed on Gallios Flow Cytometer or CytoflexS (Beckman Coulter; Brea, CA) and sorted using FACSAria (BD Bioscience, San Jose, CA) or MoFlo Astrios EQ (Beckman Coulter; Brea, CA). Data were analyzed with FlowJo software (TreeStar, Ashland, OR).

To perform cytofluorimetric analyses and cell sorting the following monoclonal antibodies (mAbs) were used: anti-CD56-PC7 and anti-NKp44-PE were purchased from Beckman Coulter (Brea, CA); anti-CD3-VioGreen (BW264/56 clone), anti-CD19-VioGreen (LT20 clone), anti-CD14-Viogreen (TÜK4 clone) mAbs were purchased from Miltenyi Biotec (Bergisch Gladbach, Germany); anti-CD45-APC-H7 (2D1 clone) from BD Biosciences (Erembodegem, Belgium); CD94-FITC, CD127-Brilliant Violet 421^TM^, CD117-PerCP/Cy5.5 were purchased from BioLegend (San Diego, CA).

### RNA Isolation and miRNA Microarray Analysis

**Figure 1:**
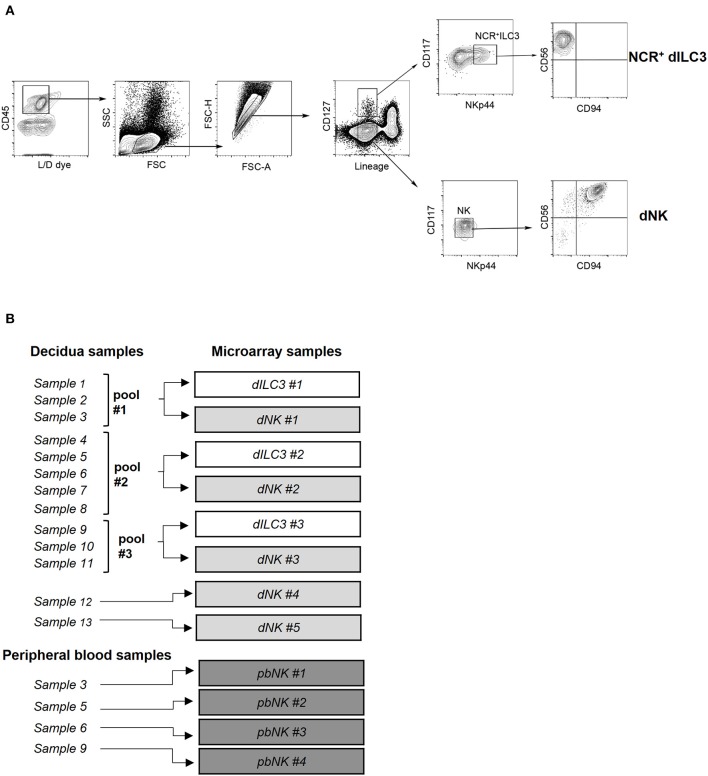
Isolation of ILC for microarray analysis. **(A)** Decidual NCR^+^ ILC3 (NCR^+^ dILC3) and decidual NK (dNK) cells were purified by cell sorting from human decidual specimens. Phenotypic characterization and gating strategy by flow cytometry of NCR^+^ ILC3 and dNK cells are shown. One representative experiment out of 13 performed. FSC, Forward scatter; SSC, side scatter. **(B)** Summary scheme of decidual and peripheral blood samples used for miRNA microarray analysis.

Total RNA extraction from purified ILCs was performed with miRNeasy micro kit following the manufacturer's protocol (Qiagen GmbH, Hilden, Germany). RNA concentration and purity was evaluated by spectrophotometric analysis (mySpec; VWR International, Radnor, PA). For pooled samples, we mixed RNA isolated from each sample following the scheme of Figure 1B. In detail, for ILC3 pool 1 we used 20 ng of sample 1, 80 ng of sample 2 and 80 ng of sample 3. The same RNA amount ratio between the samples (1:4:4) was used to create dNK pool 1. For ILC3 pool 2 and 3, we mixed equal amounts of total RNA from each sample (pool 2: 40 ng each; pool 3: 60 ng each). The same RNA amount ratio (1:1) was used to create dNK pool 2 and 3. A further check for RNA integrity was performed with 2100 BioAnalyzer (Agilent Genomics, SantaClara, CA, U.S.A.) before microarray hybridization.

The total RNA (140 ng per sample) was used as start material for the microarray based miRNA expression analysis. Biotin labeled RNA was generated using the FlashTag Biotin HSR Labeling Kit (cat#: 901910; Thermo Fisher Scientific, Wilmington, DE) according to the manufacturer's instructions. The Biotin labeled RNA was subsequently hybridized to a GeneChip™ miRNA 4.1 array plate (cat#: 902408TS; Thermo Fisher Scientific, Wilmington, DE). The hybridization, wash, staining and scanning procedures were done in a GeneTitan™ Instrument according to the manufacturer's protocol (cat#: 00-0373; Thermo Fisher Scientific, Wilmington, DE). The .cel file output (raw data files from the scanning) were used as input in the Transcriptome Analysis Console Software v 4.0.1 (TAC; Thermo Fisher Scientific, Wilmington, DE) for generation of Robust Multi-Array Average (RMA) normalized data.

Microarray data are available as GSE140606 in Gene Expression Omnibus (GEO) Database (https://www.ncbi.nlm.nih.gov/geo).

### Real Time PCR Analysis

Mature miRNA expression was assayed by miRCURY® LNA® miRNA chemistry (Qiagen GmbH, Hilden, Germany). Briefly, 20 ng of total RNA was polyadenilated and reverse transcribed with miRCURY® LNA® RT kit following manufacturer's instructions (Qiagen GmbH, Hilden, Germany). 1 μL of UniSP6 RNA spike-in (10^8^ copies/μL) was added to each sample to monitor successful reverse transcription. cDNA was diluted 1:30 in nuclease-free water and 3 μL of diluted cDNA for each replicate were loaded in 10 μL of Real Time PCR reaction mix. The remaining volume of PCR mix was composed by 5 μL of 2× miRCURY Sybr Green Master Mix (with 0.05 μL of ROX dye), 1 μL of miRCURY® LNA miRNA PCR assay, and 1 μL of nuclease-free water. The following miRCURY® LNA miRNA PCR assays were used: hsa-let-7e-5p (YP00205711), hsa-miR-125a-5p (YP00204339), hsa-miR-99b-5p (YP00205983), hsa-miR-574-3p (YP00206011), hsa-miR-146a-5p (YP00204688), hsa-miR-181a-5p (YP00206081), hsa-miR-181b-5p (YP00204530), hsa-miR-486-5p (YP00204001), hsa-miR-151a-5p (YP00204007). As endogenous controls, we used small nuclear RNA U6 (YP00203907), SNORD44 (YP00203902) and SNORA66 (YP00203905). The mean of Ct for U6, SNORD44 and SNORA66 was used to normalize miRNA expression. For mRNA quantification of lineage markers, 20 ng of total RNA was reverse transcribed with random primers by using Super Script IV first-strand synthesis system following manufacturer's instructions (Thermo Fisher Scientific, Wilmington, DE, U.S.A.). Real time PCR were carried out in 20 μL of total volume with TaqMan™ Fast Advanced Master Mix (Applied Biosystems, Foster City, CA, U.S.A). The following TaqMan™ Gene Expression assays were used: KLRD1/CD94 (Hs00233844_m1), RORC (Hs01076112_m1), CD3D (Hs00174158_m1), CD14 (Hs00169122_g1), HLA-G (Hs00365950_g1). Beta-actin (ACTB; Hs01060665_g1) was used as endogenous control. Real time PCR were carried out in triplicate on a QuantStudio 6 Flex instrument (Applied Biosystems, Foster City, CA, U.S.A). For miRNA and mRNA analysis, we used thermal PCR cycling conditions suggested by the manufacturer (Qiagen GmbH, Hilden, Germany or Applied Biosystems, Foster City, CA, U.S.A, respectively). Expression values were calculated by ΔΔCt method (with respect to dNK), or by ΔCt for lineage markers, using QuantStudio Real-Time PCR system software v. 1.3 (Applied Biosystems, Foster City, CA, U.S.A).

### Bioinformatics Analysis

**Figure 2:**
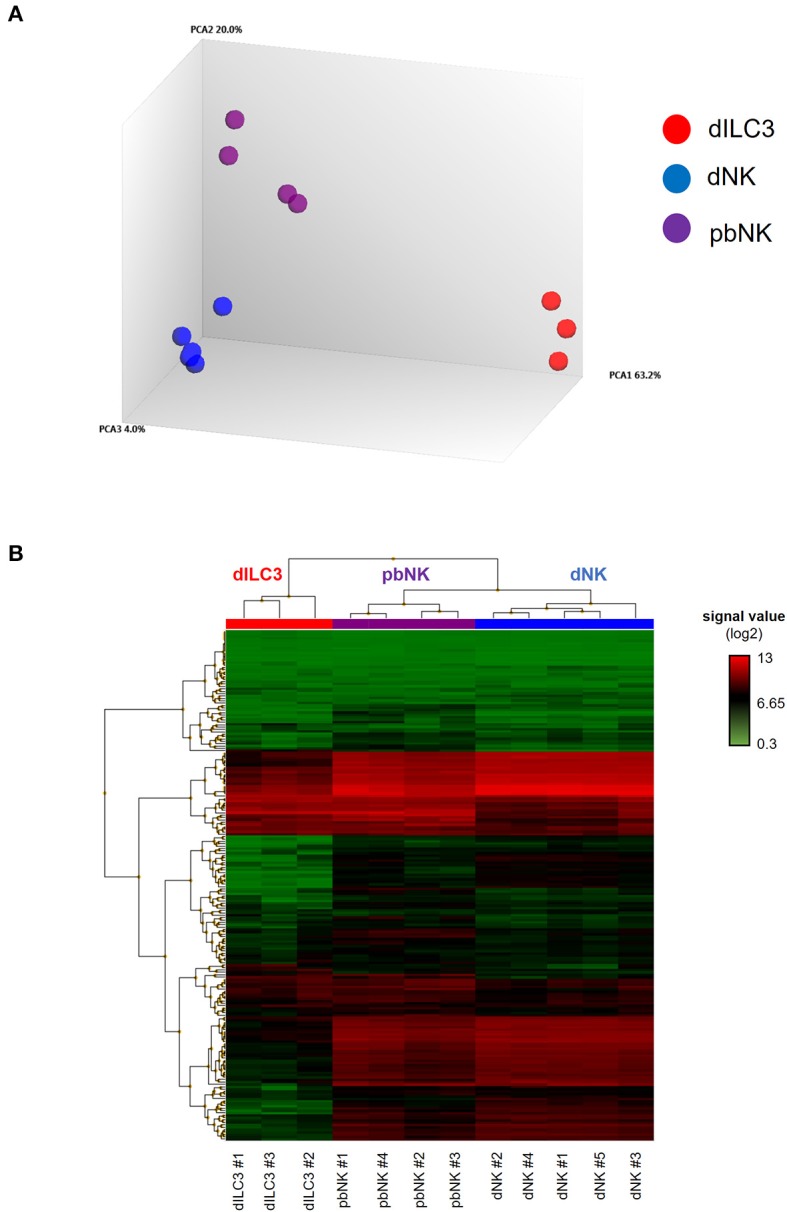
MiRNA microarray of decidual ILCs and pbNK cells. **(A)** Principal component analysis (PCA) of all the samples analyzed in the miRNA microarray. The percentages of the total variation that are accounted for by the first, second, and third principal components are shown on the x-, y-, and z-axes labels. Plots are colored by cell type; **(B)** heat-map representing the unsupervised hierarchical clustering of samples based on normalized signal values of miRNA microarray. Columns represent samples, rows represent mature miRNAs expressed above the background in all the cell types analyzed.

Principal component analysis (PCA) and the heat-map in Figure 2 were generated with TAC 4.0.1 software (Thermo Fisher Scientific, Wilmington, DE). Distance matrix plot was obtained by Exploratory Grouping analysis function of TAC software. Distance between two samples is computed using the Euclidean metric, that is, the distance is the square root of the sum of squares of the differences between equivalent signals on the samples.

Correlation scatter plot analyses between dNK, dILC3, and pbNK miRNAs were performed using the “cor” function in R v.3.5.0, adopting Pearson's correlation method. To perform Scatter plots, the “plot” function in R v.3.5.0 was used.

The identification of predicted miRNA targets was performed by TargetScan using default settings (19). The percentage of target genes shown in **Figure 6A** was calculated on the total number of human genes included in the Gene Ontology (GO) term, as obtained from AmiGO 2 search tool in the Gene Ontology Consortium database and filtered to include protein and protein complex genes type only (http://amigo.geneontology.org/) (20).

The graph shown in **Figure 6B** was generated with the target mining function of miRWalk (21). To narrow down the list to an analyzable number of interactions, we filtered the target genes selecting 3′ UTR interactions only with score ≥ 0.8 and included in miRTarbase.

Pathway enrichment analysis was performed with Panther analysis tool using Panther Pathway annotation data set (22).

### Statistical Analysis

Statistical analysis of microarray data was performed with TAC 4.0.1 software (Thermo Fisher Scientific, Wilmington, DE) using default settings for Affymetrix miRNA 4.1 library configuration. eBayes Anova method was used for limma differential expression analysis (gene level *p*-value < 0.05). A probeset is considered to be expressed if 50% of the samples in the dataset have detected above background values below the threshold (threshold *p*-value < 0.05).

Statistical analysis of real time PCR was performed with Graphpad Prism (La Jolla, CA) software. Column bars were plotted as mean and the error as standard deviation (SD); *p*-values were calculated with unpaired two-tailed Student's *t*-test. *p*-values ≤ 0.05 (^*^), ≤ 0.01 (^**^), and ≤ 0.001 (^***^) were considered statistically significant.

Statistics of pathway enrichment analysis was performed using a Fisher's exact test type with false discovery rate correction. *p*-values ≤ 0.05 were considered statistically significant.

## Results

### Isolation and Analysis of ILCs During the First Trimester of Pregnancy

Previous studies identified subsets of ILCs in human decidua during the first trimester of pregnancy (11). In order to perform a deeper comparative analysis of the predominant innate immune cells present in human decidua and of pbNK cells in pregnant women, NCR^+^ dILC3 and dNK cells were isolated from decidua basalis and NK cells from peripheral blood of healthy pregnant women undergoing voluntary termination of pregnancy in the first trimester of gestation. For isolation of dILC3 and dNK cells from decidua specimens, we applied a multiparametric cytofluorimetric gating strategy (Figure 1A). In particular, decidual leukocytes (live/CD45^+^ cells), Lineage negative (Lin^−^) (that include CD19, CD14, CD3) were gated as CD127^+^ or CD127^−^ cells. Such cell fractions were separately analyzed for the expression of suitable markers (i.e., CD117 and NKp44) allowing a more precise identification of ILC3 and NK subsets. As expected, ILC3 were included in CD127^+^CD117^+^, that could express or not NKp44. In particular, the NKp44^+^ cells represent NCR^+^ group 3 ILC and are hereinafter referred to as NCR^+^ILC3. On the contrary, CD127^−^ cells were CD117^−^NKp44^−^ and homogeneously expressed CD56 and CD94, a typical feature of dNK cells (Figure 1A). The frequency of dNK and NCR^+^ ILC3 within CD45^+^ decidual cells is reported in Supplementary Figure 1A. The purity of the sorted cell populations was confirmed by real time PCR, showing a minimal presence of lineage markers expression of other decidual cell types (Supplementary Figure 1B).

In order to evaluate a comprehensive miRNA expression profile on purified NCR^+^ dILC3 and dNK cells, a miRNA microarray analysis was performed on sorted populations isolated from human decidua samples. Due to the low number of NCR^+^ dILC3 cells obtained after sorting (average recovery ≈12,000 cells/sample), three pooled samples were analyzed (*pool #1*: samples 1–3; *pool #2*: samples 4–8; *pool #3*: samples 9–11). Further, we could include two additional non-pooled samples of dNK in the microarray. Finally, we analyzed four samples of pbNK cells derived from the same healthy pregnant women included for dNK and dILC3 analysis. A summary of the samples analyzed is reported in Figure 1B.

### Comprehensive microRNA Expression Profiling in dNK, dILC3, and pNK Cells

The miRNA microarray analysis allowed us to interrogate the expression of 2,578 mature miRNAs, 2,025 pre-miRNAs, and 1,996 of small nucleolar RNAs (snoRNAs) and small Cayal body-specific RNAs (scaRNAs) of the human genome (miRBase, release 20). We quantitatively defined the distance between each sample by a distance matrix plot, identifying three clusters according to the cell types studied (Supplementary Figure 2). Indeed, PCA revealed that dILC3, dNK and pbNK groups were clearly separated from each other's, indicating relevant differences in global miRNA expression profiles between these cell types (Figure 2A).

We decided to focus our study on the differential expression of mature miRNAs, due to their established functional role in the immune cell biology. Again, filtering the data based on mature miRNAs expressed above the background in all the cell types analyzed, the unsupervised hierarchical clustering of the samples highlighted that all of them finely clustered with respect to their cell type. Moreover, the heat map shows that miRNA profiles of dNK and pbNK hierarchically clustered together and separately from dILC3 (Figure 2B).

**Figure 3:**
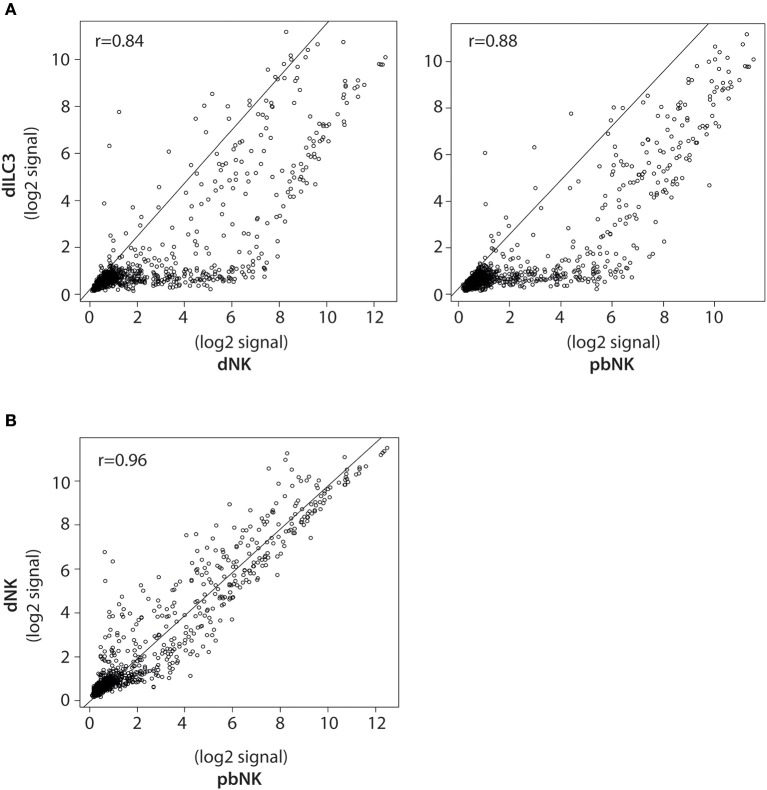
Pearson correlation analysis of miRNA expression in dILCs and pbNK cells. **(A)** Scatter plots of normalized log_2_ signal values of mature miRNAs as measured by microarray for dILC3 vs. dNK (left) or pbNK cells (right) and **(B)** dNK vs. pbNK cells. *r* = Pearson correlation coefficient.

To better compare the differences in the miRNome of dILC3, dNK, and pbNK, we used a coupled correlation analysis for the expression of all the mature miRNAs detected by the microarray, as reported in Figure 3. For all such comparative analyses, we found a significant correlation of miRNA expression levels. However, whereas pbNK and dNK miRNA expression levels were highly correlated (*r* = 0.96; Figure 3B), the difference between dILC3 and pbNK or dNK subsets resulted more apparent (*r* = 0.88 and *r* = 0.84, respectively; Figure 3A).

### Identification of Differentially Expressed microRNAs

We next focused on specific mature miRNAs differentially expressed in our samples to unveil possible epigenetic mechanisms operating in these immune cells. The comparison between microarray data of dILC3, dNK and pbNK allowed us to identify a subset of miRNAs differentially expressed in these cell types. Imposing the gene level *p*-value criterion ≤ 0.05 and the fold change (FC) criterion ≥ |2|, we found 302 differentially expressed miRNAs displaying robust and statistically significant variation between dILC3 and dNK cells. In particular, 24 miRNAs resulted up-regulated and 278 miRNAs down-regulated in dILC3 as compared to dNK cells. We also found 279 differentially expressed miRNAs between dILC3 and pbNK cells, with 10 miRNAs up-regulated and 269 down-regulated in dILC3 versus pNK cells. Finally, 155 miRNAs were significantly de-regulated between dNK and pbNK, with 77 up-regulated and 78 down-regulated in dNK as compared to pbNK. A complete list of differentially expressed miRNAs for each comparison is reported in Supplementary Tables 1–3.

**Figure 4:**
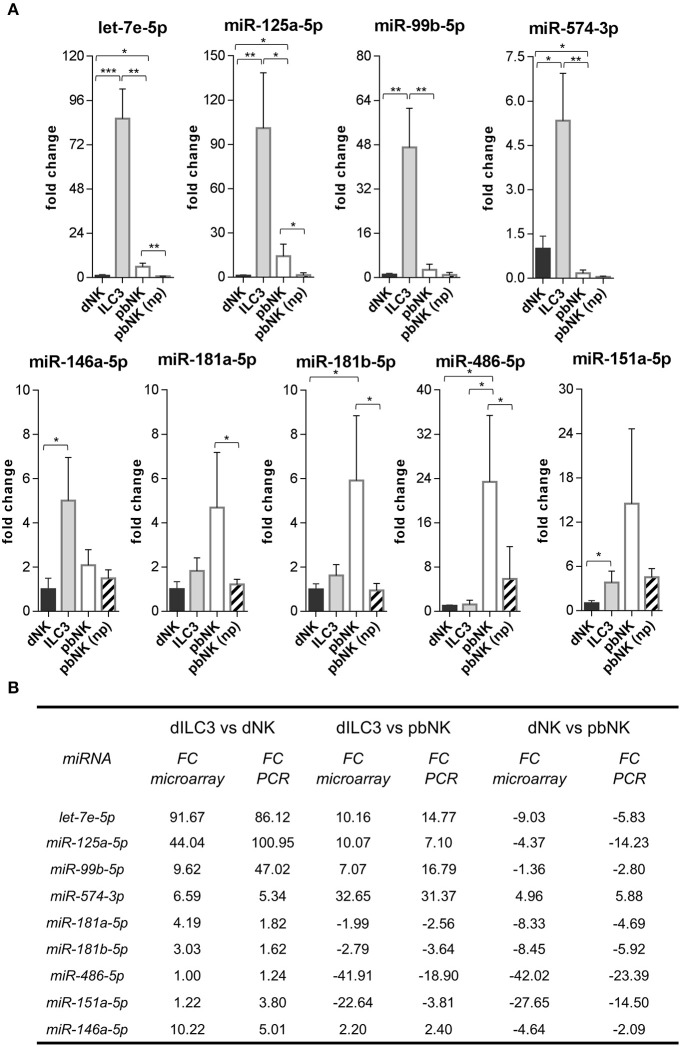
Validation of differentially expressed miRNAs evaluated by real time PCR. **(A)** The expression of the indicated miRNAs was evaluated by real time PCR in the same samples used for microarray analysis and in pNK cells derived from blood of non-pregnant (np) female controls (*n* = 4). Values are calculated with ΔΔCt method and shown as fold changes with respect to dNK. Small nuclear U6, SNORD44, and SNORA66 were used as endogenous controls to normalize miRNA expression. Histograms indicate mean values, bars indicate standard deviation (SD). *p ≤ 0.05; **p ≤ 0.01; ***p ≤ 0.001 (Student *t*-test). **(B)** Summary scheme comparing fold changes (FC) of selected miRNAs detected by real time PCR and by microarray.

Next, we decided to focus on the restricted subset of the top 10 up-regulated and down-regulated miRNAs for each comparison (Tables 1–3). To validate the microarray data, we selected from these subsets the following 9 miRNAs based on their reported function in immune cells: *let-7e-5p, miR-125a-5p, miR-99b-5p, miR-574-3p, miR-181a-5p, miR-181b-5p, miR-486-5p, miR-151a-5p, miR-146a-5p*. Then, we measured their expression by real time PCR in the samples used for microarray analysis. We also compared the miRNA levels with those of pbNK cells derived from non-pregnant female controls. We found that the expression trend across the samples of all the miRNAs tested confirmed the microarray data (Figure 4A). We observed a good concordance of FC between microarray and real time PCR, definitively confirming the reliability of the microarray data (Figure 4B). We also observed significant differences in miRNA expression between pregnant vs. non-pregnant pbNK cells. In particular, *let-7e-5p, miR-125a-5p, miR-181a-5p, miR-181b-5p*, and *miR-486-5p* resulted significantly up-regulated in pbNK of pregnant samples as compared to non-pregnant controls.

**Table 1:** Differentially expressed miRNAs in dILC3 compared to dNK cells (FC, fold change).

**microRNA**	**FC**	***p*-val**
**UP-REGULATED (TOP 10)**		
*hsa-let-7e-5p*	91.67	4.43 × 10^−08^
*hsa-miR-125a-5p*	44.04	1.06 × 10^−06^
*hsa-miR-146a-5p*	10.22	8.25 × 10^−05^
*hsa-miR-99b-5p*	9.62	5.82 × 10^−05^
*hsa-miR-4668-5p*	9.04	3.80 × 10^−03^
*hsa-miR-3613-3p*	7.95	9.40 × 10^−03^
*hsa-miR-26a-5p*	7.53	4.48 × 10^−06^
*hsa-miR-574-3p*	6.59	3.61 × 10^−05^
*hsa-miR-5189-3p*	4.51	2.00 × 10^−04^
*hsa-miR-181a-5p*	4.19	1.20 × 10^−03^
**DOWN-REGULATED (TOP 10)**		
*hsa-miR-4492*	−97.93	1.14 × 10^−08^
*hsa-miR-4505*	−81.94	1.70 × 10^−05^
*hsa-miR-3621*	−79.38	4.04 × 10^−09^
*hsa-miR-4467*	−76.11	3.75 × 10^−07^
*hsa-miR-1227-5p*	−74.18	4.67 × 10^−09^
*hsa-miR-6771-5p*	−71.16	1.74 × 10^−09^
*hsa-miR-6722-3p*	−68.77	4.96 × 10^−12^
*hsa-miR-4758-5p*	−60.63	1.30 × 10^−10^
*hsa-miR-1909-3p*	−58.05	1.12 × 10^−09^
*hsa-miR-6790-5p*	−52.65	3.46 × 10^−08^

**Table 2:** Differentially expressed miRNAs in dILC3 compared to pbNK cell (FC, fold change).

**microRNA**	**FC**	***p*-val**
**UP-REGULATED (TOP 10)**		
*hsa-miR-574-3p*	32.65	9.48 × 10^−08^
*hsa-let-7e-5p*	10.16	6.60 × 10^−05^
*hsa-miR-125a-5p*	10.07	8.63 × 10^−05^
*hsa-miR-99b-5p*	7.07	6.00 × 10^−04^
*hsa-miR-5189-3p*	3.03	2.30 × 10^−03^
*hsa-miR-3201*	2.97	4.23 × 10^−02^
*hsa-miR-4487*	2.72	4.30 × 10^−03^
*hsa-mir-320e*	2.60	3.38 × 10^−06^
*hsa-miR-4529-3p*	2.33	2.25 × 10^−02^
*hsa-mir-6722*	2.16	3.62 × 10^−07^
**DOWN-REGULATED (TOP 10)**		
hsa-miR-181a-2-3p	−66.28	1.83 × 10^−05^
hsa-miR-29a-3p	−57.53	2.39 × 10^−06^
hsa-miR-3621	−57.37	1.52 × 10^−08^
hsa-miR-3178	−54.25	3.16 × 10^−09^
hsa-miR-4492	−51.00	1.20 × 10^−07^
hsa-miR-4467	−45.02	2.19 × 10^−06^
hsa-miR-1227-5p	−42.71	3.47 × 10^−08^
hsa-miR-6771-5p	−42.65	1.07 × 10^−08^
hsa-miR-486-5p	−41.91	7.43 × 10^−08^
hsa-miR-6722-3p	−40.99	2.58 × 10^−11^

**Table 3:** Differentially expressed miRNAs in dNK cells compared to pbNK cells (FC, fold change).

**microRNA**	**FC**	***p*-val**
**UP-REGULATED (TOP 10)**		
*hsa-miR-7150*	8.60	3.61 × 10^−06^
*hsa-miR-3162-5p*	6.76	1.01 × 10^−05^
*hsa-miR-1587*	5.80	5.17 × 10^−05^
*hsa-miR-1202*	5.42	1.00 × 10^−04^
*hsa-miR-574-3p*	4.96	3.04 × 10^−05^
*hsa-miR-1229-5p*	4.93	2.00 × 10^−04^
*hsa-miR-4485*	4.76	7.00 × 10^−04^
*hsa-miR-5189-5p*	4.47	3.60 × 10^−03^
*hsa-miR-3135b*	4.44	2.72 × 10^−05^
*hsa-miR-6813-5p*	4.29	4.70 × 10^−03^
**DOWN-REGULATED (TOP 10)**		
*hsa-miR-181a-2-3p*	−70.16	4.36 × 10^−06^
*hsa-miR-486-5p*	−42.02	2.07 × 10^−08^
*hsa-miR-151a-5p*	−27.65	7.58 × 10^−09^
*hsa-miR-146b-5p*	−11.16	5.00 × 10^−04^
*hsa-miR-30b-5p*	−10.45	1.80 × 10^−03^
*hsa-miR-126-3p*	−9.27	5.33 × 10^−06^
*hsa-let-7e-5p*	−9.03	1.42 × 10^−05^
*hsa-miR-194-5p*	−8.65	5.32 × 10^−07^
*hsa-miR-29a-3p*	−8.61	9.09 × 10^−05^
*hsa-miR-181b-5p*	−8.45	5.47 × 10^−07^

### A Restricted Subset of miRNAs Is Highly Expressed in dILC3

**Figure 5:**
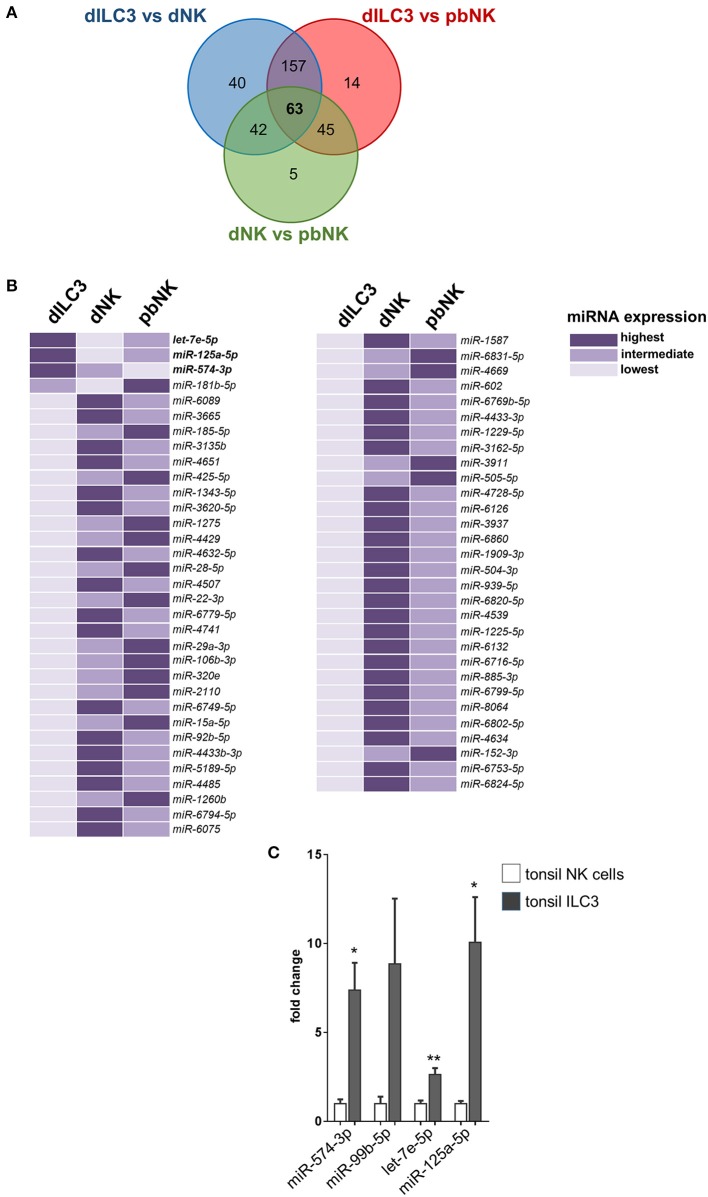
Identification of differentially expressed miRNAs in all the three comparison groups. **(A)** Venn diagram showing the number of miRNAs differentially expressed in the different comparisons between dILC3, dNK and pbNK. **(B)** List of the 63 miRNAs differentially expressed in all the comparisons, as identified by the Venn diagram. For each miRNA, the color intensity indicates the cell type with the highest (dark violet), intermediate (medium violet), and lowest (light violet) miRNA expression. **(C)** The expression of the indicated miRNAs was evaluated by real time PCR in NK cells and in ILC3 isolated from tonsils of female non-pregnant patients (*n* = 3). Values are reported as fold changes with respect to NK cells. Histograms indicate mean values, bars indicate SD. *p ≤ 0.05; **p ≤ 0.01 (Student *t*-test).

In order to identify miRNAs with relevant biological roles in the definition of ILC identity and functions, we compared in a Venn diagram the logic relations of the three sets of differentially expressed miRNAs identified by microarray (dILC3 vs. dNK, dILC3 vs. pbNK, dNK vs. pbNK; Figure 5A). With this approach, we selected 63 mature miRNAs commonly de-regulated in all the three comparison groups (Figure 5B and Supplementary Table 4). Since the expression of these miRNAs resulted significantly different among all the three cell types analyzed, we reasoned that they could be interesting candidates implicated in the specification of ILCs identity and/or functions. Notably, within this subset we found only 3 miRNAs consistently up-regulated in dILC3 as compared to dNK and pbNK (*let-7e-5p, miR-125a-5p, miR-574-3p*). In particular, *let-7e-5p* and *miR-125a-5p* expression was strikingly high in dILC3, intermediate in pbNK and low in dNK cells, whereas for the miR-574-3p the lowest expression was detected in pbNK cells (Figure 5B).

The *miR-574-3p* has been reported as an oncosuppressor miRNA in different neoplasms, inhibiting cell proliferation, migration and/or metastasis (23–25). Interestingly, *miR-574-3p* targets IL6/JAK/STAT3 pathway in hematopoietic cells (24) and TGF-β1 can up-regulate its expression (26). The miRNAs *let-7e-5p* and *miR-125a-5p* belong to the miRNA cluster *miR-99b/let-7e/miR-125a*, a genomic region embedded in the protein coding gene *SPACA6* and localized in the q13.41 on chromosome 19. Of note, the *miR-99b/let-7e/miR-125a* cluster has an important role in hematopoiesis and in the control of the cell cycle (27). Indeed, we found that *miR-99b-5p* was also strongly up-regulated in dILC3 (Figure 4), indicating that a coordinated up-regulation of the entire *miR-99b/let-7e/miR-125a* occurs in dILC3.

Furthermore, we explored the expression of miR-574-3p and the miR99b/let-7e/miR-125a cluster in ILC3 derived from a non-decidual tissue. To this purpose, we isolated by cell sorting Lin^−^CD117^+^CD127^+^ ILC3 from tonsils of three female individuals and we analyzed the expression of the miRNA subset in these cells as compared to NK cells derived from the same tissue. Remarkably, we also found a high expression of these miRNAs in tonsil ILC3 (Figure 5C), suggesting that the miRNA signature may distinguish ILC3 from NK cells beyond the decidual tissue.

### Highly Expressed miRNAs in dILC3 Target Genes Involved in the Inflammatory Response

**Figure 6:**
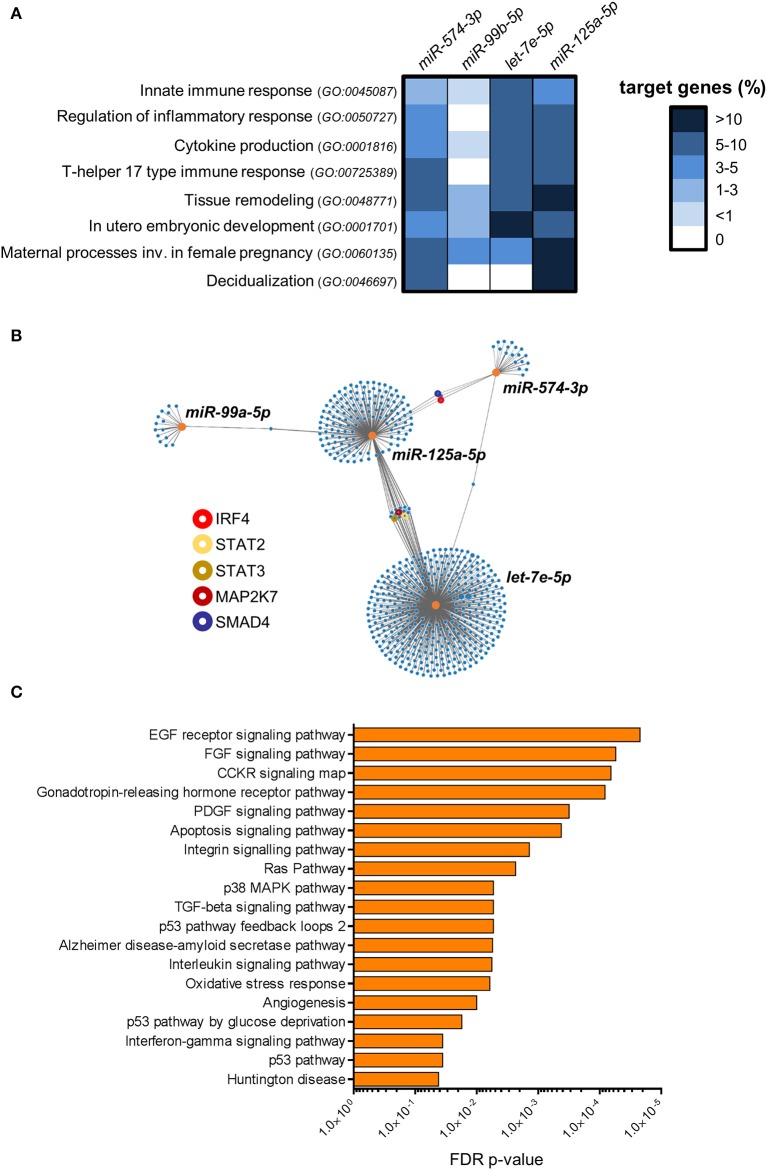
Bioinformatics analysis of miRNA targets. **(A)** The heat-map shows the percentage of genes targeted by *miR-99b-5p, let-7e-5p, miR-125a-5p* and *miR-574-3p* in the indicated GO biological processes. **(B)** Graphical representation of the network of *miR-99b-5p, let-7e-5p, miR-125a-5p* and *miR-574-3p* targets. Orange dots represent miRNAs, blue dots represent target genes. The line connections indicate experimentally validated miRNA-target interactions (from miRTarbase). Relevant target genes are highlighted by colored circles, as indicated in the legend. **(C)** Pathway over-representation analysis performed on the targets of *miR-99b-5p, let-7e-5p, miR-125a-5p* and *miR-574-3p*. Bars indicate False Discovery Rate (FDR) *p*-values. The histogram shows all the Panther pathways significantly over-represented (FDR ≤ 0.05).

To investigate the potential role of the restricted subset of miRNAs composed by *miR-574-3p* and the *miR-99b/let-7e/miR-125a* cluster in dILC3 functions, we focused on their biological targets. First, we identified predicted targets of each miRNA selecting evolutionary conserved miRNA-target interactions. Further, we implemented these target lists with the experimentally validated miRNA-target interactions registered in mirTarbase (28). Next, we decided to explore whether these miRNAs regulate specific biological processes (BPs) selected from the GO database (29, 30). We focused on BPs relevant for ILC functions, such as “Innate immune response” (*GO:0045087*), “Cytokine production” (*GO:0001816*), and “Regulation of Inflammatory response” (*GO:0050727*), since a balance between pro- and anti-inflammatory signals is critical to maintain pregnancy. We also included the BPs “T-helper 17 type immune response” (*GO:00725389*), including crucial genes for ILC3 identity and function. Finally, we selected BPs important in the physiology of decidua during the first phases of pregnancy, such as “Tissue remodeling” (*GO:0048771*), “*In utero* embryonic development” (*GO:0001701*), “Maternal processes involved in female pregnancy” (*GO:0060135*), and “Decidualization” (*GO:0046697*). For each miRNA, the percentage of genes targeted in the selected BPs were represented in the heat-map of Figure 6A. We found that the miRNA subset has a potential regulatory role in all the selected BPs. However, the analysis showed that *miR-99b-5p* did not have targets implicated in the regulation of inflammation and in T-helper 17 immune response. Conversely, *miR-125a-5p, let-7e-5p* and *miR-574-3p* hit a higher percentage of genes in the selected BPs, suggesting a major involvement in the regulation of the immune properties of ILC3. In particular, we found a high percentage of *miR-125a-5p* targets in BPs associated to female pregnancy, including decidualization (Figure 6A).

Then, we asked whether the miRNA signature could have combined regulatory effects on the same genes and molecular pathways. In Figure 6B, we represented the experimentally validated biological targets for each miRNA in a network graph. We found that the miRNA targets are largely not overlapped with those of others miRNAs, indicating that each miRNA could mainly exert regulatory effects on different set of genes. Nevertheless, we identified a small subset of genes targeted by multiple miRNAs, including important molecules in the transmission of pro-inflammatory signals in immune cells such as interferon regulatory factor 4 *(IRF4*; targeted by *miR-574-3p* and *miR-125a-5p*), Signal transducer and activator of transcription 2 (STAT2) and 3 (*STAT3*) and mitogen-activated protein kinase kinase 7 (*MAP2K7*; targeted by *let-7e-5p* and *miR-125a-5p*). In addition, SMAD family member 4 (*SMAD4*), which serves as the central mediator of TGF-β signaling, resulted targeted by both *miR-574-3p* and *miR-125a-5p* (Figure 6B).

Finally, we used the combined list of all the genes targeted by at least one selected miRNA to perform an over-representation analysis of molecular pathways (22). We identified 19 different pathways significantly enriched among the targets of the miRNA signature (FDR *p*-value ≤ 0.05; Figure 6C). Some important over-represented signaling pathways affected cell survival, proliferation, migration and/or differentiation in different cell types, such as EGF receptor, FGF, CCKR, and PDGF signaling pathways. Indeed, several target genes were implicated in a number of downstream signaling cascades connected to the above signaling pathways such as RAS, phosphatidylinositol-3 kinase (PI-3K)- protein kinase B (AKT), JAK-STAT, and several members of mitogen-activated protein kinases (e.g., MAP2K7, MAP3K1, MAPK6, etc.). Interestingly, p38 MAPK pathway, which has a central role in inflammation (31), was significantly enriched. We also found angiogenesis as a significantly over-represented pathway. Among the targets in this pathway, we found angiopoietin-2 (ANGPT2), only expressed in sites of vascular remodeling, and some members of Wnt signaling pathway (e.g., WNT1, WNT5A, FZD5) crucially involved in angiogenesis and vessel remodeling (32). Notably, in agreement with the observed targeting of the BP “Cytokine production” by the miRNAs up-regulated in dILC3 (Figure 6A), we found a significant enrichment of the interleukin and interferon-gamma signaling pathways. Among others, we found critical targets implicated in the inflammatory response targeted by the miRNA signature (e.g., IL6R, IL6, STAT3).

Intriguingly, the concomitant up-regulation of the anti-inflammatory *miR-146a-5p* in dILC3 (Figure 4) was in agreement with the existence of an up-regulated miRNA signature exerting a control on inflammatory signals in these cells.

## Discussion

In the present study, we performed a comprehensive miRNA expression profiling of ILC3 isolated from human decidua during the first trimester of pregnancy in comparison with those of decidual and peripheral blood NK cells. To our knowledge, this is the first study analyzing miRNome in human dILC3. We used cell sorting to study highly purified decidual ILC populations, focusing on NCR^+^ ILC3 that is the predominant ILC3 population in human maternal compartment (3), potentially playing important roles during the early phases of pregnancy (10). We found that distinct miRNA profiles can clearly distinguish dILC3 from dNK and pbNK cells. The global miRNA expression profiling allowed us to group all the samples according to the cell type, indicating that specific miRNA signatures can effectively characterize different ILC subsets. Nevertheless, the comparative analysis clearly revealed a high positive correlation of miRNA expressed by dILC3, dNK and pbNK cells. Thus, it is conceivable that different innate immune cells derived from a common lymphoid precursor can share some general pattern of miRNA expression in their regulatory circuits. However, whereas dNK and pbNK cells exhibited a very high correlation of miRNA profiles, dILC3 appeared more different.

MiRNAs are epigenetic modulators of gene expression in different physiological and pathological processes that occur in the endometrium (33, 34). It is known that the human placenta expresses numerous types of miRNA species, most of which grouped in in the *C14MC, C19MC* and *miR-371-3* genomic clusters (35). These pregnancy-associated miRNAs are the most expressed miRNAs in the human maternal compartments and in serum of pregnant women (35). Remarkably, the vast majority of these miRNAs was not expressed by dILC3 and dNK cells (data not shown), suggesting that the pregnancy-associated miRNA clusters are transcriptionally inactive in decidual ILCs.

Considering the subset of differentially expressed miRNAs, we focused on miRNAs displaying the highest fold changes and with well-established roles in immune cells and/or in important biological processes. Notably, we found significant differences in the miRNA expression between pregnant vs. non-pregnant pbNK cells. This is not surprising since maternal peripheral blood cells undergo significant changes during pregnancy and pbNK can acquire changes in miRNA expression due to the influence of hormonal regulation (36).

Among the miRNAs highly expressed in dILC3, we confirmed by real time PCR an increased level of *miR-146a-5p* that was recently shown to be one of the most important miRNAs in the control of inflammation and immune responses (37). The *miR-181a-5p* and *miR-181b-5p*, which were more expressed in pbNK cells, regulate innate immune cell development and function (37). We also selected *miR-486-5p* and *miR-151a-5p* which display a much higher level of expression in pbNK cells than in dILC3 and dNK cells. Interestingly, *miR-486-5p* has been proposed as a miRNA able to up-regulate the cytolytic activity of NK cells against hepatocellular carcinoma cells (38). Thus, our finding of a higher expression of *miR-486-5p* in pbNK vs. dNK cells is in agreement with the strong cytolytic activity typical of pbNK cells. The *miR-151a-5p* was found elevated in solid tumors in which it can induce cell proliferation and epithelial-mesenchymal transition (39). Of note, *miR-151a-5p* down-regulation was reported in peripheral blood mononuclear cells of psoriatic arthritis patients (40) but, so far, its potential role in NK cells is unknown. Furthermore, the three sets of differentially expressed miRNAs identified by microarray allowed us to identify a 63 miRNAs signature commonly de-regulated in all the three comparison groups. Our attention was mainly focused on dILC3. Since these cells receive a breadth of exogenous and endogenous signals of different nature in the decidual microenvironment, the identification of differentially expressed miRNAs could help understanding how these signals are simultaneously integrated in the control of ILC3 function. Three miRNAs in the 63-miRNA signature, namely *miR-125a-5p, let-7e-5p* and *miR-574-3p*, were strikingly increased in dILC3. As mentioned above, *miR-125a-5p* and *let-7e-5p* belong to the evolutionary conserved *miR-99b/let-7e/miR-125a* miRNA cluster. Interestingly, the concomitant high expression of *miR-99b-5p* suggests that the entire miRNA cluster is subjected to a strong transcriptional activation in dILC3, but not in NK cells. *Let-7e-5p* belongs to the evolutionary conserved let-7 family of miRNAs which plays a crucial role in the cell biology, including the immune system regulation (41). Notably, we showed that also others members of *let-7* are up-regulated in dILC3 as compared to dNK cells (Supplementary Table 1). The *miR-125a-5p* has an important role in hematopoiesis and in the control of the immune homeostasis (42, 43). Upon looking at the biological targets of *miR-99b/*let-7e/miR-*125a* cluster and of *miR-574-3p*, several genes were associated to selected BPs relevant for ILC functions in decidua. However, *miR-99b-5p* had a less number of targets, suggesting that the others miRNAs may play a more important role in the control of ILC3 functions. Of note, we found a high percentage of *miR-125a-5p* targets in BPs associated to pregnancy. Interestingly, the expression of *miR-125a* may be critical in pregnancy since single nucleotide polymorphism (SNPs) affecting miR-125a maturation have been associated to recurrent abortions in a Chinese population (44).

Besides the contribution of individual miRNAs to the regulation of specific BPs, we asked whether the combination of this miRNA subset could have converging regulatory roles on the same targets and/or pathways. The over-representation analysis revealed that some relevant pathways, such as angiogenesis, resulted significantly enriched. Decidual angiogenesis is a key event in the complex process of decidua formation and leukocyte recruitment. Uterine NK cells have strong angiogenic potential and play an essential role in normal early decidual angiogenesis (45). Our data revealed that an epigenetic control of angiogenesis mediated by this restricted signature of highly expressed miRNAs can also occur in dILC3. Furthermore, we found several target genes involved in the positive transmission of pro-inflammatory stimuli in the over-represented interleukin and IFN-γ signaling pathways, such as *IL6R* and *STAT3*. Notably, although the majority of target genes was not shared by different miRNAs of the subset, we found some important genes with implication in the inflammatory response simultaneously targeted by *miR-125a-5p* and *let-7e-5p* or *miR-574-3p*. Importantly, the *miR-99b/let-7e/miR-125a* cluster has been shown to have anti-inflammatory function in immune cells (46). Further, *miR-125a* promotes the fundamental shift from inflammation to immune suppression by controlling regulatory T cells homeostasis (43). Some evidence suggests that *miR-574-3p* may also have anti-inflammatory function (24). Overall, the presence of several target genes implicated in the regulation of the inflammatory response suggested that the highly expressed *miR-125a-5p, miR-574-3p*, and *let-7-5p* subset may play a role in dampening the pro-inflammatory activity of dILC3. Successful pregnancy requires an early state of mild inflammation, which favors tissue remodeling and trophoblast migration necessary for implantation. However, an uncontrolled inflammatory response could lead to pregnancy failure. In line with our findings, a decreased expression of let-7 members, included let-7e-5p, has been reported in aborted decidua samples (47). We could also find signaling pathways associated to cell survival, proliferation, migration and differentiation that are significantly over-represented within the miRNA targets. Thus, it is tempting to speculate that the miRNA signature, dampening pro-proliferative and inflammatory signals, could also have a role in limiting ILC3 expansion in human decidua. ILC3 are dependent on molecular signals received via pro-survival cytokines allowing their maintenance. Increased proportions of ILC3s have been found in the decidua of women undergoing spontaneous preterm labor (48). Thus, a local regulation of such cells may be required to maintain a successful pregnancy. Further studies, aimed to investigate the expression of the identified miRNA signature in decidual ILC3 from recurrent miscarriage, may help to better clarify the role of these miRNAs in the maintenance of pregnancy.

Our data revealed that miR-574-3p and the miR-99b/let-7e/miR-125a cluster were also highly expressed in tonsil ILC3. This finding indicates that the miRNA signature may also characterize ILC3 different from decidual tissue. Thus, these miRNAs could represent an epigenetic mark of ILC3 not specifically induced by the decidual microenvironment. However, further research is needed to confirm this hypothesis.

In this study, the function of the miRNA signature identified is inferred by bioinformatics analysis. However, further investigation, such as the modulation of miRNA expression in ILCs and/or in fetal trophoblast cells, is required to confirm the functional role of these miRNAs in the decidua and in other tissues. Nevertheless, the data herein reported increases the body of knowledge on ILC3 cell biology and provides a starting point for future research, aimed at identifying new epigenetic mechanisms implicated in the regulatory network of innate immune cells in human decidua.

In conclusion, here we have characterized for the first time the miRNome of ILC3 in human decidua, showing that specific miRNA profiles distinguish dILC3 from dNK and pbNK cells. We also identified a restricted signature of miRNAs in dILC3 representing interesting candidates for a potential role as a molecular brake of exaggerated proliferation and inflammatory responses during the early phases of pregnancy.

## Data Availability Statement

The datasets generated for this study can be found in the Gene Expression Omnibus database (accession number GSE140606).

## Ethics Statement

The studies involving human participants were reviewed and approved by the ethics committee of Azienda Ospedaliera Universitaria San Martino, IRCCS Policlinico San Martino Hospital, Genoa, Italy. The patients/participants provided their written informed consent to participate in this study.

## Author Contributions

AP designed and performed research, interpreted data, and wrote the article. CA performed research and wrote the article. TI processed and analyzed tonsil samples. FL and NT performed flow cytometry and sorting experiments. MM and LM interpreted data and revised the manuscript. PV designed research, interpreted data, and wrote the article.

### Conflict of Interest

The authors declare that the research was conducted in the absence of any commercial or financial relationships that could be construed as a potential conflict of interest.
