# Supplementary figures and images for: An Anti-inflammatory microRNA Signature Distinguishes Group 3 Innate Lymphoid Cells From Natural Killer Cells in Human Decidua
Source: Front Immunol. 2020 Feb 6;11:133. doi: 10.3389/fimmu.2020.00133 (PMC7015979; doi:10.3389/fimmu.2020.00133)

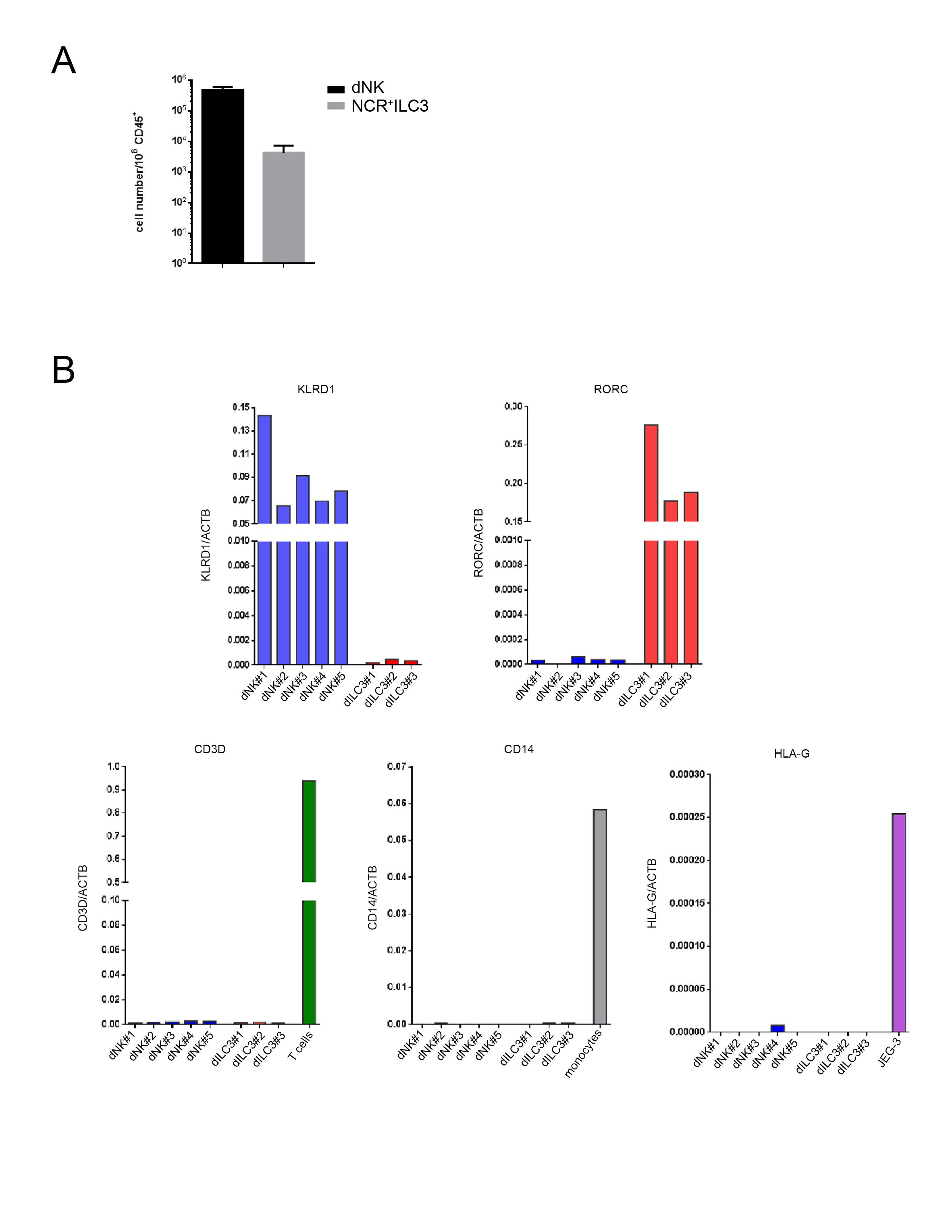

Supplement: Supplementary file 5 [file Image_1.TIF]

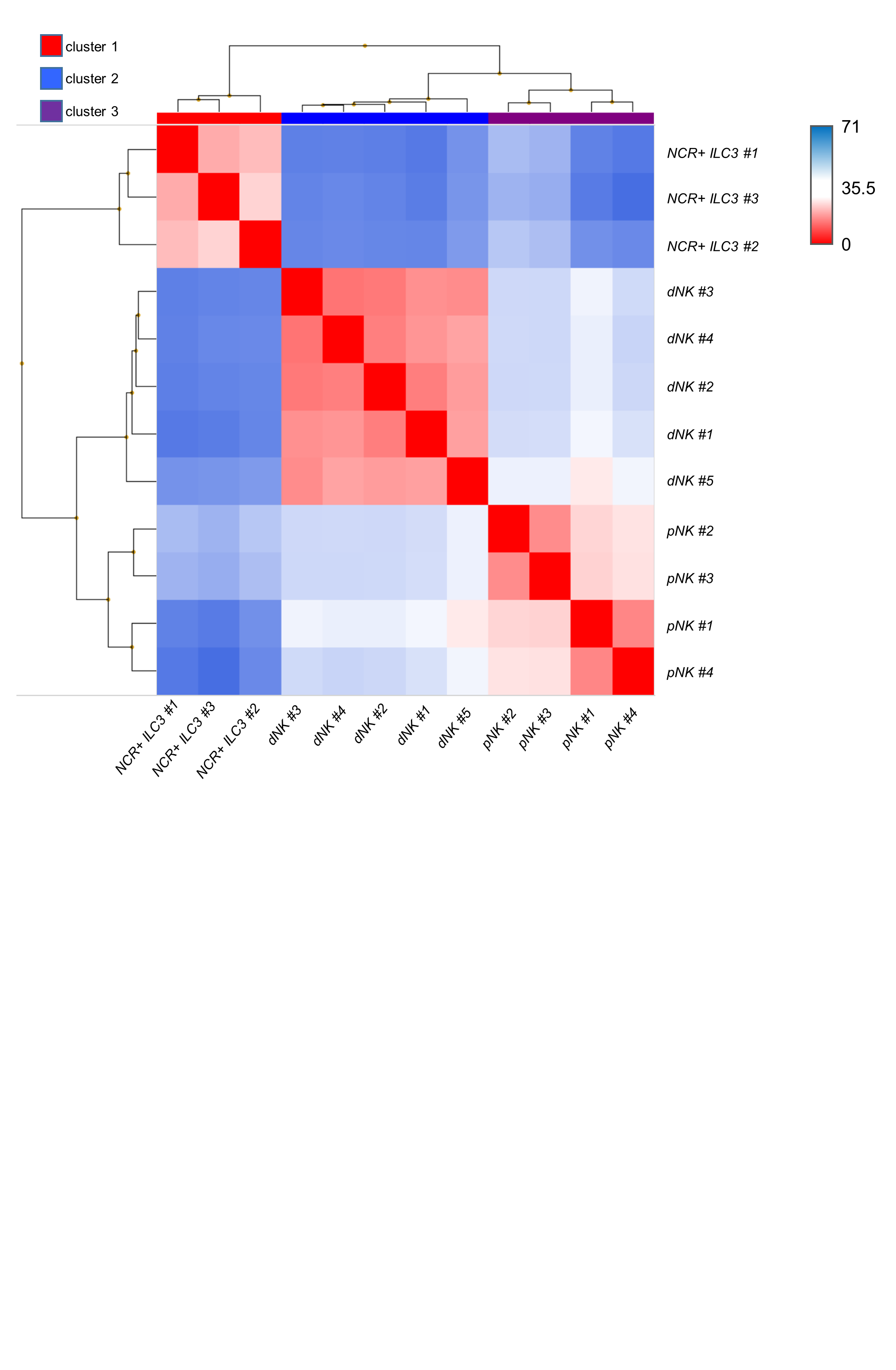

Supplement: Supplementary file 6 [file Image_2.TIF]
