# Supplementary material for: An Anti-inflammatory microRNA Signature Distinguishes Group 3 Innate Lymphoid Cells From Natural Killer Cells in Human Decidua
Source: Front Immunol. 2020 Feb 6;11:133. doi: 10.3389/fimmu.2020.00133 (PMC7015979; doi:10.3389/fimmu.2020.00133)
Supplement: Supplementary file 7 [file Data_Sheet_1.pdf]

Supplementary Figure 1. Frequency and purity of sorted decidual cells. **(A)** Frequency of dNK and NCR+ ILC3 in decidual samples was measured by flow cytometry and expressed as cell number on 10^6 $CD45^+$ decidual cells; **(B)** Real time PCR analysis on the decidual samples used in the microarray for KLRD1/CD94, RORC and the lineage markers of T cells (CD3D), macrophage (CD14) and trophoblast (HLA-G) cells. As positive controls, pan T cells and monocytes were isolated from blood with Pan T and Pan Monocyte Isolation kit, respectively (Miltenyi Biotech, Bergisch Gladbach, Germany). The human choriocarcinoma cell line JEG-3 was kindly provided by P. Le Bouteiller (Unit INSERM, Toulouse, France). Beta-actin (ACTB) was used as endogenous control. Values are calculated with ΔCt method.

Supplementary Figure 2. Distance matrix of microarray samples. The matrix graph of microarray samples quantitatively showing the distance between the samples. Dark red indicates no distance between the samples. Samples with different expression profiles are colored in blue. The three clusters identified NCR^+ dILC3 (red), dNK (blue) and pbNK (violet) samples.
